# Therapeutic Appropriateness of Cariprazine in the Management of Schizophrenia: Experts’ Opinion using a Delphi Approach

**DOI:** 10.2174/1570159X21666230719162023

**Published:** 2023-09-01

**Authors:** Bernardo Dell’Osso, Antonello Bellomo, Andreas Pietro Maria Conca, Virginio Salvi, Alberto Siracusano, Carmelo Zaffora, Domenico De Berardis, Massimo Di Giannantonio

**Affiliations:** 1 Department of Mental Health and Addiction, University of Milan, ASST Fatebenefratelli-Sacco, Milan, Italy; "Aldo Ravelli" Center for Nanotechnology and Neurostimulation, University of Milan, Milan, Italy; Department of Psychiatry and Behavioral Sciences, Stanford University, Stanford, CA, USA;; 2 Department of Clinical and Experimental Medicine, Psychiatric Unit, University of Foggia, Foggia, Italy;; 3 NHS, National Health Service, Department of Mental Health, Bolzano, Italy;; 4 Auxiliary University Department, Medical University of Innsbruck and Paracelsus Medizinische Privatuniversität (PMU), Salzburg, Austria;; 5 Department of Clinical Neurosciences/DIMSC, Università Politecnica delle Marche, Ancona, Italy;; 6 Psychiatric Clinic, PTV Foundation - Policlinico Tor Vergata University of Rome, Rome, Italy;; 7 NHS, National Health Service, Department of Mental Health, Azienda Sanitaria Provinciale (ASP 3), Catania, Italy;; 8 NHS, National Health Service, Department of Mental Health, ASL 4, Teramo, Italy;; 9 Department of Neuroscience, Imaging and Clinical Sciences, University "G. d'Annunzio" of Chieti, Pescara, Chieti, Italy

**Keywords:** Schizophrenia, cariprazine, positive symptoms, negative symptoms, metabolism, quality of life, switch

## Abstract

**Background:**

Schizophrenia is a psychiatric disorder whose therapeutic objectives are aimed at reducing symptoms and improving patient’s quality of life. First- and second-generation antipsychotics present numerous side effects. Recently introduced in the treatment of schizophrenia, cariprazine has shown to improve positive and negative symptoms as well as cognitive impairment, with good tolerability.

**Objective:**

To assess the level of consensus among Italian psychiatrists in relation to the use of cariprazine in the treatment of schizophrenia by using the Delphi technique.

**Methods:**

A Delphi study was undertaken between January and July 2022. Two questionnaires were consecutively sent to a panel of 97 psychiatrists from all over Italy, of which 81 actively participated, anonymously, in at least one of the two consultations with a sufficiently high response rate (83%).

**Results:**

Broad consensus in terms of the efficacy and safety of cariprazine in the treatment of schizophrenia during all phases of the disorder. The young first-episode schizophrenia patient with or without substance abuse seems to be an excellent candidate for cariprazine therapy. In addition, the lack of side effects makes cariprazine a suitable drug for adult and elderly patients with schizophrenia. However, there is still limited experience with the use of cariprazine, along with little knowledge of the most recent real-life data.

**Conclusion:**

These results could encourage wider dissemination of evidence-based practices with the final aim of optimizing the clinical use of cariprazine in patients with schizophrenia.

## 1 INTRODUCTION

Schizophrenia (SCZ) is a psychiatric disorder with onset in adolescence/early adulthood, characterized by potentially rapid cognitive deterioration since the earliest stages of the disease [1]. The symptomatology includes positive symptoms such as delusions, hallucinations, disorganized speech/ thinking, and negative symptoms such as social isolation, anhedonia, emotional flattening, abulia, and avolition [1, 2]. Negative symptoms, in particular, have a strong impact on quality of life (QoL) [3, 4].

Recently updated International Guidelines for the treatment of schizophrenia assess pharmacological management and treatment through the various stages of the disease, including the first episode, relapse prevention, and conditions that have been refractory to standard treatment [5]. In particular, the guidelines of the American Psychiatric Association (APA) recommend a careful evaluation of the patient in terms of a review of psychiatric symptoms and history of trauma [6]. Ultimately, shared therapeutic objectives include clinical remission of the acute phase, prevention of psychotic recurrences and preservation of cognitive reserve, control of negative symptoms in the late stages of the disease, functional recovery, and management of any side effects of antipsychotic drugs and medical comorbidities [1].

Antipsychotics of different chemical classes are the first-line drug treatments for SCZ [7, 8]. First- and second-generation antipsychotics act on D2 dopamine receptors and have a significant effect on the balance between receptors, by acting as 5HT2A serotonergic receptors antagonists [1, 9].

These classes of antipsychotics allow good symptom control but may cause extrapyramidal side effects and metabolic disorders, such as hyperprolactinemia and disorders of glucose metabolism [1, 9]. Third-generation antipsychotics are considered, by some psychiatrists, to be the first line in the treatment of SCZ [10]. Among these, cariprazine presents an innovative mechanism of action aimed at improving treatment not only of positive symptoms, but also of negative symptoms, often underestimated, and of cognitive reserve, with good tolerability [1]. It is a piperazine derivative [11] that has been approved by the European Medicines Agency (EMA) and the United States Food and Drug Administration (FDA) for the treatment of SCZ [12].

Cariprazine differs from all other atypical antipsychotics as it presents a greater affinity to the D3 receptor than dopamine itself [13], thus exhibiting functional D3 receptor partial agonism in the human brain that other antipsychotics do not possess [12, 14, 15].

Cariprazine can be prescribed as an adjunct medication to drugs such as clozapine, when antipsychotics in monotherapy are ineffective for negative symptoms [16], with a recommended dosage of 1.5-6 mg/day [12].

Cariprazine, an atypical antipsychotic, at lower dose range (from 1.5 to 3 mg/day) has shown to act as a dopamine-serotonin partial agonist, also displaying antidepressant and procognitive effects. At an intermediate dose range (from 3 to 4.5 mg/day) acts as a typical antipsychotic, being effective in treating the positive and negative symptoms of schizophrenia. Finally, at high doses (4.5-6 mg/day), cariprazine is a potent D2 receptor partial agonist, almost like risperidone, a typical antipsychotic drug [17-19].

In addition to managing symptoms, treatment with cariprazine can offer patients a better QoL, in terms of autonomy and greater access to psychosocial rehabilitation pathways [9]. Despite the innovative profile of cariprazine, in terms of efficacy and low side effects, it is not known how the molecule is perceived and used by Italian psychiatrists.

Based on these considerations, our study was aimed at clarifying current clinical practice and strategies to be implemented for the effective management of adult patients (>18 years) with SCZ, with reference to the use of cariprazine. The ultimate purpose was to reach an agreement on a standard treatment that could be applied in daily clinical practice, and an assessment of the knowledge of the use of cariprazine in real life, by evaluating the level of consensus among a representative group of Italian psychiatrists.

## 2 METHODS

The Delphi approach provides a consensus-based strategy that, through the sharing of opinions, professional experience, and scientific evidence, aims to achieve agreement on the proposed topics [20].

This study was conducted between January and July 2022, among Italian psychiatrists with a relevant experience in the treatment of patients with SCZ. A modified two-round Delphi method was used. The study was divided into three phases: exploratory, analytical, and evaluative.

### 2.1 Exploratory Phase

After identifying the objectives, Menthalia S.r.l., a provider of scientific and medical communication, identified a Scientific Coordinator for the project and a Board composed of 7 psychiatrists working in University and Hospital Institutions or local psychiatric services (*i.e*., the authors of the current study). Psychiatrists selected from throughout Italy were required to have the following qualifications: clinical experience in the treatment of patients with SCZ, active participation in committees of the Italian Society of Psychiatry and other scientific societies, and authorship of multiple international or national publications on the topic of SCZ.

After sharing the objectives, and under the supervision of the Scientific Coordinator, an in-depth bibliographic search was conducted to identify 4 macro-areas of investigation and to develop a semi-structured questionnaire. The questionnaire was then submitted to the members of the Board for critical validation and draft improvement to create a subsequent questionnaire for the first Delphi round.

The final version of the first questionnaire was then submitted to the Experts Panel, consisting of psychiatrists working in Italian hospitals and local psychiatric services. The Panel of Board-certified pediatricians was selected from a database of Menthalia S.r.l by identifying 400 Italian psychiatrists with scientific requirements similar to those of the Board members: relevant clinical experience, participation in scientific societies, publications on the topic of SCZ (at least 5 publications in the last 5 years), different background.

Of the 400 psychiatrists identified by Menthalia S.r.l., 97 responded spontaneously, confirming their will to participate in the study. The 97 volunteer experts were sent a first email with a brief description of the survey, its purpose, and the proposed methodology.

It should be noted that, at present, in a modified Delphi survey, there is no gold standard for the size of the sample. In fact, experts believe in purposive sampling rather than probability sampling. Therefore, the panel member selection was not based on the number of clinicians but followed a specific procedure driven by criteria of competence, knowledge, and experience [21].

A literature search was performed by Menthalia S.r.l. with the following search strings: guidelines AND schizophrenia management, schizophrenia management AND partial agonist, second-generation antipsychotics *versus* first-generation antipsychotics, atypical *versus* typical antipsychotics, third-generation antipsychotics, cariprazine AND positive symptoms, cariprazine AND hostility, cariprazine AND negative symptoms, cariprazine AND switch, cariprazine AND metabolism, cariprazine AND quality of life, cariprazine AND cardiovascular.

The research only considered studies carried out on the adult population (>18 years) and published in the last 5 years. No distinctions were made regarding the type of study. An analysis of the titles and abstracts of all the results obtained from the bibliographic research was carried out, and the appropriateness of the contents with respect to the objective was assessed. Redundant studies, off-topic studies, and reviews that synthesized studies already selected and summarized in this document or that expressed concepts already underlined were eliminated. Regarding the comparison of cariprazine with other antipsychotics, only direct comparative studies or systematic reviews conducted for the same purpose were selected. The results obtained were framed into 4 macro-areas, a useful format for the study design, represented by:

• Prescriptive appropriateness in the management of adult patients (>18 years) with SCZ (third-generation antipsychotics *vs*. first- and second-generation antipsychotics);

• Efficacy and tolerability of cariprazine *versus* other antipsychotics (including studies *versus* placebo);

• Appropriateness of the dosage range in the switch from other antipsychotics to cariprazine;

• SCZ and QoL.

### 2.2 Analytical Phase

Based on bibliographic research, the first semi-structured questionnaire, consisting of 21 items divided into 4 macro-areas (Table **1**), was formulated and administered to the Board on March 11, 2022.

Levels of agreement/disagreement for each statement were quantified by a 5-point Likert scale (1, *strong disagreement*; 2, *fair disagreement*; 3, *no opinion*; 4, *fair agreement*; 5, *strong agreement*); the average score and the count were reported considering statements valid when answers converged for 70% of cases in the 2 highest levels of consensus.

With reference to the multiple-choice questions, the count of the preferences expressed for each option was reported.

Having the Board shared, reviewed, and approved, the semi-structured questionnaire, the Experts Panel was involved in the first Delphi round by sending them the final version of the first questionnaire.

### 2.3 Evaluation Phase

The first Delphi questionnaire, consisting of 23 statements divided into 4 areas, was administered anonymously to the panel of 97 experts through a certified web platform. The panel was provided with a bibliography supporting the statements.

Levels of agreement/disagreement for each statement were measured by a 5-point Likert scale (1, *strong disagreement*; 2, *fair disagreement*; 3, *no opinion*; 4, *fair agreement*; 5, *strong agreement*); at the end of each section, an “Other” line was provided to allow voters to justify their decision. For each statement, the average score and the percentage of voters who gave positive responses were considered, and the cut-off level for consensus was 70% agreement.

The consultation took place between April 11, 2022 and May 10, 2022 and, during the same period, 3 reminders were sent to the panel to acquire the widest possible participation. After the first round of consultations, the discussion led to the reformulation of 5 statements. The Board processed the answers given by the panelists and produced a second questionnaire, which was eventually submitted to the same panel of experts.

The second modified questionnaire, consisting of 23 statements divided into 4 sections, was once again administered to the panel of 97 experts with the same method used in the first round. The consultation took place between May 26 and June 15, 2022.

## 3 RESULTS

The Delphi questionnaire was sent to 97 psychiatrists from all over Italy: of them, 81 actively participated in at least one of the 2 consultations with a sufficiently high response rate (83%).

Of the 97 experts who declared their willingness to participate, 81 completed the questionnaire in full. In general, in a Delphi consultation, assertions that reach an agreement of at least 70% on the 2 most favorable positions are considered approved.

For a more correct evaluation of the answers, the mean score and index of dispersion were also used. The results of the first and second Delphi rounds are summarized in Tables **2a**, **b**, **c**, and **d** (Table **2a-d**).

It should be noticed that, at the end of the first Delphi round, statements 3, 5 and 6 of the first section, statement 15 of the second section, and statement 23 of the third section did not obtain an adequate average score and were characterized by a high index of dispersion. For this reason, they were revised.

At the end of the second Delphi round, statement 15 of the second section and statement 23 of the third section were validated, whereas statements 3, 5 and 6 once again did not obtain an adequate average score despite the revision performed at the end of the first Delphi round.

## 4 DISCUSSION

The main finding of the study was that the psychiatrists who participated in the two Delphi rounds agreed with most of the statements proposed by the Board in the two questionnaires. An amendment to some of the statements was required, and it is precisely on those statements that the Board’s discussion was focused during the final meeting held remotely on July 5, 2022 (*via* the Zoom platform).

### 4.1 First Macro-Area: Therapeutic Appropriateness in The Management of The Adult Patients (> 18 Years) with SCZ

Most of the statements in this area achieved validation in the first Delphi round. In particular, the team of psychiatrists agrees in arguing that the therapeutic choice is subject to the assessment of the disease status [5, 6] and that second-generation antipsychotics are to be preferred in terms of therapeutic results. As for cariprazine, the Experts Panel agreed that this drug, due to its mechanism of action on D3 receptors [22], improves cognitive symptoms, and that its use could be very useful in young patients with short disease duration. There was also agreement on the minimum dose to be used for cariprazine (1.5 mg), in accordance with the literature [1, 12, 16], and the Board agreed that cariprazine is a drug capable of reducing craving and, therefore, can be used in patients with a history of substance abuse.

On the contrary, there was no agreement, neither in the first nor in the second Delphi round, on statements 3, 5 and 6.

As for the partial efficacy shown by first- and second-generation antipsychotics over negative symptoms of SCZ (statement 3), the reformulation of the statement did not improve the dispersion indicator; rather agreement on the two highest levels of judgment (4, *fair agreement; 5, strong agreement*) was reduced. Against this assertion, which had been the subject of extensive discussion since the beginning, non-homogeneous judgments with a high dispersion index were expected, as it embodies a reference to the entire class of first- and second-generation antipsychotics; ultimately, it was not conceivable to add more specific assertions on this topic.

The cut-off was not reached even after the reformulation of statement 5, relating to the use of cariprazine in psychotic onset with hostile behavior. In this case, an additional literature search on the efficacy of cariprazine in early psychotic-onset patients with hostility was requested, which added only one study [23] to the initial selection. In general, from the discussion, it seemed that, in the treatment of patients with early psychotic onset and hostility, there is a tendency to prefer the use of drugs that allow the therapeutic benefit to be achieved quickly at low doses, such as clozapine, whereas for cariprazine the titration process recommended by the Summary of product characteristics may require some time.

Statement 6, relating to the use of cariprazine (also in combination with a second drug) in psychotic episodes with motor agitation, also did not reach the cut-off. It should be noted that, in this case, specifying that the second drug could have also been administered intramuscularly could have favored greater consensus. In fact, psychiatrists who are not greatly confident using cariprazine may prefer to administer an antipsychotic monotherapy such as aripiprazole or olanzapine intramuscularly, which has good therapeutic results on motor agitation at label doses. At the end of the discussion, there was agreement on the same conclusions made for the previous statement, or that, in the presence of motor agitation, an initial dosage of 1.5 mg of cariprazine may not be quickly effective, even in combination with another drug. Therefore, those who are not confident using the drug and have little knowledge of clinical practice data do not seem to use cariprazine.

### 4.2 Second Macro-Area: Switch from One Antipsychotic to Another

Most of the statements in this area achieved validation in the first Delphi round. The Experts Panel agreed on switching from one antipsychotic to another in the event of the onset of side effects and/or inadequate therapeutic response [24, 25]. As for switching from another antipsychotic to cariprazine, the experts agreed on the validity of cross-tapering and the usefulness of a switch in case of metabolic symptoms, typical side effects of second-generation antipsychotics [1, 26].

Statement 15, relating to the switch to cariprazine in elderly (over-65-year-old) patients with SCZ-spectrum disorders for whom good margins of functional recovery are identifiable, was reformulated for the second consultation, which led to an improvement in all statistical indicators and to reaching the cut-off. Literature reports efficacy and safety data from the use of cariprazine in patients over the age of 65, in the approved daily dosage range for adults of 1.5-6 mg [27]. Despite the data, the Board believed that additional literature searches on this topic would be appropriate as, usually, these patients often have a history of chronic schizophrenia and multiple treatments, and not all doctors agree on administering a new molecule in order to try to obtain recovery, functional and potential benefits.

### 4.3 Third Macro-Area: Treatment Safety and Tolerability

All the statements in this area received validation in both the first and the second Delphi rounds. Experts agreed that treatment with cariprazine is associated with a lower risk of QT interval prolongation [28] and a low risk of metabolic disorders and hyperprolactinemia [29].

### 4.4 Fourth Macro-Area: SCZ and QoL

In this fourth area, most of the statements reached consensus since the first Delphi round, with the exception of statement 23.

Statement 23, relating to the use of cariprazine both as a drug of first choice in patients with SCZ-spectrum disorders and as a long-term treatment, was then further discussed. In this case, several literature studies support the use of cariprazine as a long-term therapy, with good tolerance to treatment in terms of adverse effects and long-term metabolic complications [22, 29, 30].

The subsequently modified statement reached the cut-off, with no disagreeing opinions. Instead, neutral judgment was widely used, likely indicating that a communication and information campaign about the appropriate use of the drug would be beneficial to illustrate its potential both among first choices and in the long-term, as already reported in the literature [16].

## CONCLUSION

The results of this Delphi survey show a broad consensus on the overall efficacy and safety of cariprazine in the treatment of schizophrenia during all phases of the disorder. The young first-episode schizophrenia patient with or without substance abuse seems to be an excellent candidate for cariprazine therapy. In addition, the safety profile of cariprazine and especially the lack of metabolic, sexual, and cardiac side effects make it a drug of the first choice, especially, but not only, in first-episode youth. The lack of these side effects makes cariprazine a suitable drug for adult and elderly patients with schizophrenia. In addition, the receptor profile, especially partial D3 agonism, by improving cognitive and negative symptoms, also reflexively enhances the patient's quality of life, and studies in the literature demonstrate this. Switching to cariprazine also presents no particular problems.

On the other hand, results show that there is still limited experience with the use of cariprazine within the Italian psychiatric community, and a lack of knowledge of the most recent real-life evidence.

In fact, the clinical practice highlights how the recommended starting dosage of cariprazine (1.5 mg once a day) is not readily effective in “difficult-to-treat” schizophrenic patients with hostility and/or motor agitation, with or without onset psychosis. This implies that these patients do not benefit from treatment with cariprazine, although there are encouraging data in the literature regarding its use at higher dosages [1, 12, 16]. This may be due to the fact that cariprazine at a lower dosage recommended by the data sheet may have an 'activating' effect that is useful in more inhibited patients but may be perceived by psychiatrists as a problem in patients with severe hostility and aggressive behaviour. In these cases, in everyday 'real-world' clinical practice, one often starts directly with at least a 3 mg dosage. Often in these patients, there is also the practice of concomitantly using antipsychotic drugs with a higher D2 blockade when increasing cariprazine to 3 mg would suffice. In any case, one can start cariprazine at the recommended dosage of 1.5 mg concomitantly with another potent D2-blocking antipsychotic, progressively reducing the doses of the latter and increasing cariprazine as per the drug's datasheet.

Additionally, the importance of further studies on the population of over-65-year-old patients with chronic SCZ should be noted, especially with regard to life extension, because at present, as reported by the Board, these patients are mainly treated by geriatricians, with limited focus on psychiatric aspects.

In conclusion, the take for future actions is that a much greater level of communication and information on the optimal use of cariprazine is needed, together with wider dissemination of evidence-based practices.

Finally, we emphasize that this Delphi survey gives opinions about the present therapeutic management of schizophrenia. Therefore, these results may be subject to future modifications based on updates from the use of this new drug on the market.

## Figures and Tables

**Table 1:** Semi-structured questionnaire administered to the Board.

**A**	**Therapeutic Appropriateness in the Management of Adult Patients (>18 Years) with SCZ**	
**1**	In the management of a schizophrenic patient, the evaluation of the stage of the disease is decisive for the therapeutic choice.	[Scale 1-5]
**2**	Second-generation antipsychotics should be preferred over first-generation antipsychotics as they are associated with better functional outcomes and less cognitive impairment.	[Scale 1-5]
**3**	In schizophrenic patients with predominantly negative symptoms, first- and second-generation antipsychotics have shown unsatisfactory or inadequate efficacy.	[Scale 1-5]
**4**	Cariprazine, in addition to affecting negative and positive symptoms by acting on D3 receptors, also produces an improvement in cognitive symptoms (attention, mood, concentration) facilitating psychosocial rehabilitation treatments.	[Scale 1-5]
**5**	In the case of a schizophrenic patient with the early psychotic onset and with positive symptoms, including hostility, the use of a third-generation antipsychotic, such as cariprazine, is more appropriate.	[Scale 1-5]
**6**	In the case of patients with SCZ-spectrum disorders and agitation (in combination with a second psychotropic drug, *e.g*., benzodiazepine), the use of cariprazine is more appropriate.	[Scale 1-5]
**7**	In acute SCZ associated with positive and negative symptoms, cariprazine may be useful, especially in young patients with relatively short disease duration.	[Scale 1-5]
**8**	In patients with SCZ-spectrum disorders and a history of substance abuse, cariprazine is particularly suitable as it can reduce craving.	[Scale 1-5]
**9**	It is always appropriate to start early treatment with cariprazine using the minimum recommended dosage of 1.5 mg once a day.	[Scale 1-5]
*9a*	If you disagree, give reasons	-
**B**	**Switch From one Antipsychotic to Another**	-
**10**	The lack of a significant therapeutic response and the appearance of side effects deemed unacceptable for the clinician and/or the patient are the main reasons behind the decision to switch from one antipsychotic to another.	[Scale 1-5]
**11**	In the absence of objective necessity, especially if not carried out in the hospital the timeframe to complete the switch to cariprazine from another antipsychotic until complete titration of the drug is about 3 weeks.	[Scale 1-5]
12	Cross-tapering is the most suitable method for switching to cariprazine, to avoid dopaminergic, histaminergic, or cholinergic rebounds.	[Scale 1-5]
*12a*	If you disagree, give reasons	-
**13**	**Switching to cariprazine may be particularly useful for patients with SCZ who:**	-
	- Have a partial response to the current antipsychotic, especially to negative or cognitive symptoms - Have partial adherence - May show fewer side effects with cariprazine than with the parent compound (*e.g*., hyperprolactinemia, sedation or metabolic effects) - Show comorbid substance abuse - Other	Select one or more options
*13a*	*Other (specify)*	-
**14**	**In the elderly diagnosed with chronic SCZ, switching to cariprazine may be particularly useful in patients:**	-
	- With an SCZspectrum for which good margins of functional recovery are identifiable - With comorbidities (cardiopathic, oncological, metabolic, *etc*.) - At suicidal risk - With an SCZ-spectrum disorder and a history of substance abuse - Other	Select one or more options
*14a*	***Other (specify)***	-
**C**	**Treatment Safety and Tolerability**	-
**15**	In the choice of antipsychotic therapy, the importance of limiting any adverse events that may create stigmatization for the patient (extrapyramidal symptoms, weight gain, sexual dysfunction, sedation) should be taken into account.	[Scale 1-5]
**16**	Compared to most first- and second-generation antipsychotics, cariprazine is associated with a lower risk of weight gain, metabolic disturbances, insulin resistance and dyslipidemia.	[Scale 1-5]
**17**	Compared to most first- and second-generation antipsychotics, cariprazine is associated with a lower risk of QT interval prolongation.	[Scale 1-5]
**D**	**SCZ and QOL**	
**18**	In the management of the patient with SCZ, the medium- and long-term therapeutic goal has shifted from symptom control to the patient’s functional recovery.	[Scale 1-5]
**19**	Recognizing and monitoring cognitive symptoms, together with negative symptoms, represent the most significant obstacle to the patient’s good relationship with the social world and, therefore, to their quality of life.	[Scale 1-5]
**20**	The greater effect on negative symptoms, which condition the quality of life and functional abilities of the patient, allows a valid predisposition to rehabilitation programs for family, social, and work reintegration.	[Scale 1-5]
**21**	Long-term treatment with cariprazine can prevent relapse in schizophrenic patients, thus suggesting that it can be used as first-line therapy.	[Scale 1-5]

**Note:** Scale: 1, strong disagreement; 2, fair disagreement; 3, no opinion; 4, fair agreement; 5, strong agreement, **Abbreviation**: SCZ: Schizophrenia; QoL: Quality of life.

**Table 2a:** Summary results of the two Delphi consultations.

-		**First Round**			**Second Round**		
		**Mean Score**	**Agreement on 4 and 5**	**Index of ** **Dispersion**	**Mean Score**	**Agreement on 4 and 5**	**Index of Dispersion**
A	**Therapeutic Appropriateness in the Management of Adult Patients (>18 Years) with SCZ**	-	-	-	-	-	-
1	In the management of a schizophrenic patient, the evaluation of the stage of the disease is decisive for the therapeutic choice.	4.4/5	92.9%	33.0%	4.5/5	94.2%	30.3%
2	Second-generation antipsychotics should be preferred over first-generation antipsychotics as they are associated with better functional outcomes and less cognitive impairment.	4.5/5	95.7%	28.6%	4.6/5	95.7%	28.3%
4	Cariprazine, in addition to affecting negative and positive symptoms by acting on D3 receptors, also produces an improvement in cognitive symptoms (attention, concentration) facilitating psychosocial rehabilitation treatments.	4.1/5	85.7%	33.4%	4.1/5	88.4%	30.9%
7	In acute SCZ, associated with positive and negative symptoms, cariprazine could be useful, especially in young patients with relatively short disease duration.	4.2/5	88.6%	34.9%	4.2/5	89.9%	35.5%
8	Cariprazine is appropriate in patients with SCZ-spectrum disorders and substance abuse as it can reduce cravings.	3.9/5	71.4%	38.7%	4.0/5	81.2%	35.1%
9	It is advisable to start treatment with cariprazine using the minimum recommended dosage of 1.5 mg once a day.	4.2/5	82.9%	44.4%	4.3/5	91.3%	38.3%
3	*In schizophrenic patients with predominantly negative symptoms, first- and second-generation antipsychotics have shown partial efficacy.*	3.7/5	71.4%	59.9%	Statement modified for the second consultation		
3	First- and second-generation antipsychotics have shown partial efficacy in countering the negative symptoms of SCZ.	Statement modified from the first consultation			3.7/5	69.6%	60.6%
5	*In schizophrenic patients with early psychotic onset with hostility, the use of cariprazine is appropriate.*	3.3/5	51.4%	60.4%	Statement modified forthe second consultation		
5	The use of cariprazine is appropriate in psychotic beginnings with hostile behavior.	Statement modified from the first consultation			3.4/5	49.3%	56.8%
6	*In patients with SCZ-spectrum disorders and agitation * *(also in combination with a second drug, e.g., benzodiazepine) the use of cariprazine is appropriate.*	3.5/5	60.0%	55.8%	Statement modified for the second consultation		
6	The use of cariprazine (also in combination with a second drug) is appropriate in psychotic episodes with motor agitation.	Statement modified from the first consultation			3.6/5	60.9%	56.3%

**Table 2b:** Summary results of the two Delphi consultations.

-		**First Round**			**Second Round**		
		**Mean Score**	**Agreement on 4 and 5**	**Index of ** **Dispersion**	**Mean Score**	**Agreement on 4 and 5**	**Index of Dispersion**
B	**Switch from one Antipsychotic to Another**	-	-	-	-	-	-
10	The lack of a significant therapeutic response and the appearance of side effects are the main reasons behind the decision to switch from one antipsychotic to another.	4.7/5	100%	20.4%	4.7/5	98.6%	23.1%
11	On an outpatient basis, the timeframe to complete the switch to cariprazine from another antipsychotic until complete titration of the drug is approximately 3 weeks.	3.9/5	77.1%	40.8%	3.9/5	85.5%	37.2%
12	Cross-tapering is the most suitable method for switching to cariprazine, to avoid dopaminergic, histaminergic, or cholinergic rebounds.	4.4/5	92.9%	31.6%	4.5/5	97.1%	27.7%
13	Switching to cariprazine may be useful for patients who have a partial response to negative or cognitive symptoms.	4.4/5	95.7%	30.3%	4.4/5	94.2%	30.5%
14	Switching to cariprazine may be useful for patients who have fewer side effects with cariprazine than with the parent compound (*e.g*., hyperprolactinemia, sedation, or metabolic effects).	4.2/5	87.1%	39.3%	4.5/4	95.7%	29.1%
16	In the elderly over 65 diagnosed with chronic SCZ, switching to cariprazine may be useful in patients with comorbidities (cardiopathic, oncological, metabolic, *etc*.).	4.0/5	78.6%	40.0%	4.0/5	79.7%	41.0%
15	*In the elderly over 65 diagnosed with chronic SCZ, switching to cariprazine may be useful in patients with an SCZ-spectrum for whom good functional recovery margins are identifiable.*	3.7/5	70.0%	41.4%	Statement modified for the second consultation		
15	Switching to cariprazine may be useful in elderly patients over 65 with schizophrenia-spectrum disorders in whom good margins of functional recovery are identifiable.	Statement modified from the first consultation			3.8/5	73.9%	35.0%

**Table 2c:** Summary results of the two Delphi consultations.

-		**First Round**			**Second Round**		
		**Mean Score**	**Agreement on 4 and 5**	**Index of Dispersion**	**Mean Score**	**Agreement on 4 and 5**	**Index of Dispersion**
C	**Safety and Tolerability of Treatment**	-	-	-	-	-	-
17	In the choice of antipsychotic therapy, it is important to limit the adverse events that may create stigmatization for the patient (extrapyramidal symptoms, weight gain, sexual dysfunction, sedation).	49./5	98.6%	12.6%	4.9/5	100.0%	6.7%
18	Compared to most first- and second-generation antipsychotics, cariprazine is associated with a lower risk of weight gain, metabolic disturbances, insulin resistance and dyslipidemia.	4.4/5	90.0%	34.0%	4.6/5	95.7%	26.8%
19	Compared to most first- and second-generation antipsychotics, cariprazine is associated with a lower risk of QT interval prolongation.	4.2/5	82.9%	41.5%	4.6/5	94.2%	29.3%

**Table 2d:** Summary results of the two Delphi consultations.

-		**First Round**			**Second Round**		
		**Mean Score**	**Agreement on 4 and 5**	**Index of Dispersion**	**Mean Score**	**Agreement on 4 and 5**	**Index of Dispersion**
D	**SCZ and QoL**	-	-	-	-	-	-
20	In the management of the schizophrenic patient, the medium- and long-term therapeutic goal has shifted from symptom control to the patient’s functional recovery.	4.7/5	98.6%	21.2%	4.7/5	97.1%	25.4%
21	Recognizing and monitoring cognitive symptoms, together with negative symptoms, represent the most significant obstacle to the patient’s good relationship with the social world and therefore to their quality of life.	4.5/5	90.0%	35.8%	4.5/5	95.7%	30.8%
22	The greater effect on negative symptoms, which condition the quality of life and functional abilities of the patient, allows a sure predisposition to rehabilitation programs for family, social, and work reintegration.	4.6/5	100.0%	23.3%	4.6/5	97.1%	25.0%
23	*Based on clinical data, cariprazine is indicated both as * *first-choice and long-term treatment.*	3.9/5	68.6%	47.5%	Statement modified for the second consultation		
23	Based on clinical data, cariprazine is indicated both as a drug of first choice in patients with SCZ-spectrum disorder and as a long-term treatment.	Statement modified from the first consultation			4.0/5	73.9%	41.3%

**Abbreviations:** SCZ: Schizophrenia; QoL: Quality of life.

## Data Availability

Not applicable.

## LIST OF ABBREVIATIONS

APA: American Psychiatric Association
EMA: European Medicines Agency
FDA: United States Food and Drug Administration
QoL: Quality of Life
SCZ: Schizophrenia
